# Peripheral Neuropathy in Children and Adolescents with Insulin-dependent Diabetes Mellitus

**Published:** 2018

**Authors:** Nosrat GHAEMI, Hossein HASANABADI, Farah ASHRAFZADEH, Somaye SARVARI, Hamidreza RAHIMI, Somayyeh HASHEMIAN

**Affiliations:** 1 Department of Pediatric Endocrinology, Faculty of Medicine, Mashhad University of Medical Sciences, Mashhad, Iran; 2 Department of Physical Medicine, Faculty of Medicine, Mashhad University of Medical Sciences, Mashhad, Iran; 3 Department of Pediatric Neurology, Faculty of Medicine, Mashhad University of Medical Sciences, Mashhad, Iran; 4 Department of Pediatrics, Faculty of Medicine, Mashhad University of Medical Sciences, Mashhad, Iran; 5 5-Department of Modern Sciences and Technology, Faculty of Medicine, Mashhad University of Medical Sciences, Mashhad, Iran

**Keywords:** Type 1 diabetes mellitus, NCV, Diabetic peripheral neuropathy, HbA1c level

## Abstract

**Objective:**

Type 1 diabetes mellitus (T1DM) is a chronic immune-mediated disease. Diabetic peripheral neuropathy (DPN) is an important microvascular complication of T1DM. One of the most important risk factors for the development of DPN is poor glycemic control. We evaluated the prevalence of DPN among T1DM patients and determined the association between DPN and glycated hemoglobin (HbA1c) level.

**Materials & Methods:**

The subjects were recruited prospectively upon initial evaluation at Imam Reza Hospital, Mashhad, Iran from Jan 2013 to Jan 2015. Patients with T1DM were selected based on the inclusion criteria (i.e., age of 6≤yr and absence of other co-morbidities). DPN was assessed through electrodiagnostic studies and neurological examinations, while diabetes control was evaluated by measuring the HbA1c level.

**Results:**

Fifty patients with T1DM were enrolled in this study. The mean diabetes duration of patients was 8.38±3.79 yr (mean age16.68±6.68 yr). The mean HbA1c level was 8.6±2.1% in patients without DPN and 10.5±3 in those with DPN (*P*=0.016). Overall, 24% of the subjects were presented with DPN according to nerve conduction velocity study (NCV) findings. A positive correlation was found between NCV and clinical symptoms with signs (*P*<0.001, r=0.45 and *P*<0.001, r=0.644, respectively). Sensitivity and specificity of neurological examination for DPN diagnosis were 91.7% and 63.2%, respectively. Poor diabetes control is associated with DPN. Moreover, HbA1c level was used as an index for glycemic control over the past 3 months.

**Conclusion:**

Rigid blood glucose control and periodic neurological examinations were the best strategies to prevent DPN.

## Introduction

Type 1 diabetes mellitus (T1DM) is a chronic immune-mediated disease, in which the pancreas ceases to produce enough insulin (1). The overall increase in the incidence of T1DM was estimated at nearly 3%, and the annual incidence of this condition varied from 0.61 cases per 100000 populations in China to 57.6 cases per 100000 in Finland (2, 3). Middle East region, Saudi Arabia and Kuwait have the highest incidence rate at 31.4 and 22.3/100000 per year (4).

Annually, 1 to 35 new cases of T1DM (younger than 14 yr) per 100000 populations are diagnosed per year. In various countries, the incidence rate of T1DM has at least doubled over the past two decades. The incidence of this condition seems to raise as the distance from the equator increases (5, 6). 

Peripheral nervous system involvement is a common complication of T1DM, and unlike adults, patient’s polyneuropathy has no clinical manifestations in diabetic children (7). The prevalence of neuropathy varies depending on the severity and duration of hyperglycemia (1). The prevalence of peripheral neuropathy in both adults and children ranges between 7% and 57% (8).

Nerve conduction velocity (NCV) test is the method of choice for the diagnosis of neuropathy. The incidence of neuropathy in different studies ranges from 10% to 68% among diabetic patients (9, 10). 

In this study, we evaluated the sign and symptoms of peripheral polyneuropathy and its correlation with glycated hemoglobin (HbA1c) level as an indicator of metabolic control in diabetic patients in Mashhad City, Eastern Iran.

## Materials & Methods

 The subjects were recruited prospectively upon initial evaluation at Imam Reza Hospital, Mashhad, Iran from Jan 2013 to Jan 2015. Patients with T1DM were selected based on the inclusion criteria, i.e., age of six years or older, and absence of other comorbidities. On the other hand, patients with neurological disorders, such as mental retardation, secondary diabetes, systemic diseases, or maturity-onset diabetes of the young were excluded from the study.

Ethics Committee of Mashhad University of Medical Sciences approved this study. Written consent forms were obtained from all study subjects. 

Anthropometric data including body mass index (BMI), weight, and height were measured while recruiting the participants. HbA1c level and lipid profile were analyzed by applying the immune turbid metric method (BT3000 analyzer, Italy). An expert neurologist and endocrinologist to determine DPN was carried out a detailed neurological examination. Vibration perception, temperature sensation, pinprick sensation, MMT and ankle reflex were also examined in the participants. A tuning fork (128 Hz), Semmes-Weinstein monofilament test, cold and warm water test, and a reflex hammer were used for the evaluations. Neuropathy was evaluated through electrodiagnostic studies. The reference for evaluation and comparison of the electro-diagnostic parameters of sensorimotor responses were based on the tables from the “Electrodiagnosis in diseases of the nerve and muscle” and “electro-diagnostic medicine”. The evaluation was done in a calm room with optimal lighting and temperature was at 24-degree Celsius. Patients' skin surface temperature was approximately 34 degrees Celsius, at the time of evaluation.

The sensory nerves evaluated were the median and ulnar nerves in the upper limbs, Sural and superficial peroneal nerves in the lower limbs, and sural as well as superficial peroneal nerves in lower limbs. The methods used for the stimulation, recording, and analysis of the data were based on the standards of the books aforementioned.

NCV and distal latency tests were performed in the following nerves: Common peroneal nerve, tibial nerve, superficial peroneal nerve, and sural (sensory) nerve in the lower extremities, as well as median (motor and sensory) nerves in the upper extremities, were observed in the tests. Electrodiagnostic studies were conducted by one single operator and device (Myto, Italy). We evaluated 50 diabetic patients, divided into two groups: group A with abnormal NCV, and group B with normal NCV.

Normality of variables was tested by Kolmogorov–Smirnov test. Then, parametric or non–parametric tests were performed to determine the difference and relationship between variables. For data analysis, SPSS Ver. 16 (Chicago, IL, USA) was applied, and the collected data with referral codes were imported into the software. *P*-value less than 0.05 were considered statistically significant. 

## Results

FiftyT1DM patients with the mean age of 16.68±6.98 yr and disease duration of 8.36±3.79 yr were enrolled (Table 1). Overall, 54% (n=27) of the participants were female. Twenty one (42%) patients were clinically asymptomatic, while 29 (58%) cases had at least one of the signs or symptoms of peripheral neuropathy. Abnormal pain sensation was detected in 9 (18%) patients, and the frequency of paresthesia, numbness, and burning sensation was 6%, 28%, and 8%, respectively; in total, and 30 (60%) patients had one abnormal symptom. Neurological examination was suggestive of impaired temperature sensation in 6 (12%) cases. Reduced vibration perception, abnormal Achilles reflex, and impaired touch perception were reported in 5 (10%), 15 (30%), and 3 (6%) cases, respectively; overall, 29 (58%) patients had one abnormal sign. Abnormal Achilles reflex and numbness were the most common findings in our study population (Table 2).

**Table1:** Clinical and biochemical characteristics of diabetic patients with and without neuropathy

**Risk factors**	**Neuropathy**		***P*** **-value**
	**Present**	**Absent**	
Sex (male/female)	3/9	20/18	0.094
Age (yr)	19.7±10.1	15.7±5.4	0.20
Diabetes duration (years)	11.3±9.4	7.4±3.7	0.18
BMI (kg/m^2^)	21.1±2.6	19.7±3.3	0.16
Triglyceride (mg/dl)	105.6±66.5	83.7±44.7	0.35
Cholesterol (mg/dl)	158.33±34.8	188.7±64.4	0.28
HbA1c	10.58±3.05	8.63±2.1	0.016

Data are presented as Mean±SD

BMI: Body mass index; TG: Triglyceride; TC: Total cholesterol**,** HbA1c: Glycated hemoglobin

**Table 2:** Frequency of signs and symptoms in diabetic patients according to nerve conduction velocity (NCV

		**Abnormal NCV** **N=12 (24%)**	**Normal NCV** **N=38(76%)**	**Total** **N=50**
Symptoms	Burning sensation	3 (25)	1(2.6)	4 (8)
	Pain sensation	3 (25)	6(15.8)	9 (18)
	Paresthesia	2 (16)	1(2.6)	3 (6)
	Numbness	5 (41.7)	9 (23.7)	14 (28)
**Signs**	Impaired temperature sensation	6 (50)	0 (0)	6 (12)
	Reduced pain sensation	0 (0)	0 (0)	0 (0)
	Reduced vibration perception	5 (41.7)	0 (0)	5(10)
	Impaired touch perception	3(25)	0 (0)	3(6)
	Abnormal Achilles reflex	9 (75)	6 (15.8)	15 (30)

**Table 3:** The correlation between clinical indices and nerve conduction velocity (NCV)

	**Conduction**	**Normal NCV** **N=38(76%)**	**Abnormal NCV** **N=12 (24%)**	**Kappa coefficient**
**Abnormal signs**	**Absent**	24 (63.2)	3 (25)	Kappa=0.290 *P*-Value=0.021
	**Present**	14 (36.8)	9 (75)	
	**Total**	38 (100)	12 (100)	
**Abnormal symptoms**	**Absent**	32 (84.2)	1 (8.3)	Kappa=0.664 Sig<0.001
	**Present**	6 (15.8)	11(91.7)	
	**Total**	38 (100)	12 (100)	

Based on the comparison between the two groups by Kappa coefficients, clinical signs were not significantly associated with peripheral neuropathy, while a significant correlation was found between peripheral neuropathy and clinical symptoms (Table 3). NCV was normal in 38 (76%) patients and abnormal in 12(24%) cases. The mean HbA1c level was 8.6±2.1 (mmol/ml) in patients without peripheral neuropathy, and 10.5±3 in those with peripheral neuropathy (*P*=0.016) (Table 1).

## Discussion

Diabetic neuropathy is a major complication of T1DM. This term usually refers to polyneuropathy and can be classified into two broad subclinical and clinical stages. The prevalence of diabetic neuropathy in developing countries ranges between 10% and 68% (9,11). In Western countries, up to 60% of all patients with diabetes are affected by diabetic neuropathy (10). However, the estimated rates of children are inaccurate, considering the prevalence of subclinical diabetic neuropathy observation in this age group.

In the present study, DPN was reported in 24% of the patients, according to the electrodiagnostic studies, while 25% of these cases were clinically asymptomatic. Nearly one-fourth of young patients who recently diagnosed with diabetes had pathological findings in the distal nerves (9). Moreover, subclinical DPN was reported in up to 60% of patients in Isfahan, Iran (12). Various studies have introduced diabetes duration as a major factor in DPN development (11, 13-16), although this finding has not been confirmed in other studies (17, 18), similar to the present research. On the other hand, diabetic neuropathy and abnormal NCV findings have been reported in patients with recently diagnosed diabetes by some researchers (14, 19). 

Patients with diabetic neuropathy have few complaints, and their physical examination may reveal variable degrees of sensory loss (20). In our study, 28% of all subjects experienced numbness and 30% showed abnormal tendon reflex. Reduction in pain perception on neurological examinations, abnormal tendon reflex, and numbness were more significant in-group A, compared to group B. The frequency of abnormal pain sensation, paresthesia, and burning sensation was estimated at 18%, 6%, and 8%, respectively. Neurological examination was suggestive of impaired temperature sensation, reduced pain perception, reduced vibration perception, abnormal tendon reflex, and impaired touch perception in 12%, 0%, 10%, 30%, and 6% of subjects, respectively. 

The present findings revealed a positive correlation between clinical symptoms and abnormal NCV with sensitivity of 91.7% and specificity of 84.2% (Table 3). Therefore, serial neurological examinations could be used for the diagnosis of diabetic neuropathy in clinics. Deep peroneal and sural nerves were the most commonly affected nerves in our patients (18); moreover, in two patients, the mentioned diagnosis associated with carpal tunnel syndrome was detected. 

Although hyperglycemia plays a crucial role in the progression of DPN, DPN can also occur without hyperglycemia (21, 22). The 24-yr prospective study of 32 newly diagnosed patients with type 1 diabetes and comparing well controlled with poorly controlled patients, showed that near- normoglycemic control from the diagnosis over a 24 yr was associated with a strong prevention of peripheral neuropathy development (23).

Our results revealed that poor diabetes control could be associated with DPN, and HbA1c level, used as an index for glycemic control over the past six months. A previous study has suggested an association between hyperlipidemia and DPN (24); however, in Jaiswal .M et.al study have not confirmed this correlation, similar to the present study(25). The prevalence of diabetic peripheral neuropathy was 7% in youth with type 1 diabetes and risk factors were older age, longer diabetes duration, increased blood pressure, obesity, increased LDL cholesterol and triglycerides (25). In fact, in the present study, diabetic neuropathy had no positive correlation with age, sex, BMI, duration of disease, or hyperlipidemia, whereas a significant correlation between HbA1c level and diabetic neuropathy was observed.

**In conclusion, **the best strategy to prevent diabetic neuropathy is rigid blood glucose control and periodic neurological examinations and the poor glycemic control is a determining factor in peripheral neuropathy in type 1 diabetic patients.
